# *PhERF2*, an ethylene-responsive element binding factor, plays an essential role in waterlogging tolerance of petunia

**DOI:** 10.1038/s41438-019-0165-z

**Published:** 2019-07-01

**Authors:** Dongmei Yin, Daoyang Sun, Zhuqing Han, Dian Ni, Ayla Norris, Cai-Zhong Jiang

**Affiliations:** 1 College of Ecology, Shanghai Institute of Technology, Shanghai, 201418 China; 2 College of Landscape Architecture and Arts, Northwest A&F University, Yangling, Shaanxi 712100 China; 3 Crops Pathology & Genetic Research Unit, United States Department of Agriculture, Agricultural Research Service, Davis, CA 95616 USA; 4 Department of Plant Sciences, University of California Davis, Davis, CA 95616 USA

**Keywords:** Flooding, Plant molecular biology

## Abstract

Ethylene-responsive element binding factors (ERFs) are involved in regulation of various stress responses in plants, but their biological functions in waterlogging stress are largely unclear. In this study, we identified a petunia (*Petunia* × *hybrida*) ERF gene, *PhERF2*, that was significantly induced by waterlogging in wild-type (WT). To study the regulatory role of *PhERF2* in waterlogging responses, transgenic petunia plants with RNAi silencing and overexpression of *PhERF2* were generated. Compared with WT plants, *PhERF2* silencing compromised the tolerance of petunia seedlings to waterlogging, shown as 96% mortality after 4 days waterlogging and 14 days recovery, while overexpression of *PhERF2* improved the survival of seedlings subjected to waterlogging. *PhERF2*-RNAi lines exhibited earlier and more severe leaf chlorosis and necrosis than WT, whereas plants overexpressing *PhERF2* showed promoted growth vigor under waterlogging. Chlorophyll content was dramatically lower in *PhERF2*-silenced plants than WT or overexpression plants. Typical characteristics of programmed cell death (PCD), DNA condensation, and moon-shaped nuclei were only observed in *PhERF2*-overexpressing lines but not in *PhERF2*-RNAi or control lines. Furthermore, transcript abundances of the alcoholic fermentation-related genes *ADH1-1*, *ADH1-2*, *ADH1-3*, *PDC1*, and *PDC2* were reduced in *PhERF2*-silenced plants, but increased in *PhERF2*-overexpressing plants following exposure to 12-h waterlogging. In contrast, expression of the lactate fermentation-related gene *LDH* was up-regulated in *PhERF2*-silenced plants, but down-regulated in its overexpressing plants. Moreover, PhERF2 was observed to directly bind to the *ADH1-2* promoter bearing ATCTA motifs. Our results demonstrate that *PhERF2* contributes to petunia waterlogging tolerance through modulation of PCD and alcoholic fermentation system.

## Introduction

Global climate change brings about a frequent occurrence of extreme rainfall events, and it increases the demand for improvement of plant tolerance to waterlogging^1^, which is defined as the state in which soil is saturated with water most of the time, restricting exposure to air and causing anaerobic conditions. Plants under waterlogging conditions undergo hypoxic stress with difficulty in oxygen diffusion, resulting in a drop in photosynthesis, respiration, and chlorophyll accumulation^2^. Adaptation of plants to low oxygen levels takes place at three stages^3^. At the beginning, several signal transduction components are rapidly induced in plants, and then metabolic adaptation is initiated through fermentation pathways. Finally, programmed cell death (PCD) and cell wall autolysis cause morphological changes such as aerenchyma formation in adventitious roots^4^. PCD is closely related with the cellular phenomenon of DNA condensation and moon-shaped nuclei^5^. These molecular and morphological adaptations serve to promote oxygen retention and capture efficiencies for alleviating waterlogging stress.

Plants produce metabolic energy through fermentative glycolysis and not oxidative respiration in response to waterlogging stress^6–8^. In the case of oxygen deficiency, respiration varies from the aerobic to the anaerobic mode, which is implicated in glycolysis and fermentation^9^. The first step of fermentation pathway reveals that pyruvate decarboxylase (PDC) is responsible for catalysis of the conversion of pyruvate to acetaldehyde. Then acetaldehyde is converted to ethanol via alcohol dehydrogenase (ADH), leading to regeneration of NAD^+^ in dehydrogenase systems. This process plays a major role in the alcoholic fermentation pathway, and is vital for sustainment of glycolysis under hypoxic conditions^10^. Besides, the conversion of pyruvate to lactate by lactate dehydrogenase (LDH) also contributes to the production of NAD^+^. The functional characterization of anaerobic proteins (ANPs), such as PDC, ADH, and LDH, have been reported in some plant species, including Arabidopsis^11^, salt marsh grass^12^, rice^9^, pigeon pea^10^, and maize^13^.

The ethylene-responsive element binding factor (ERF) proteins are crucial transcriptional regulators in response to diverse biotic and abiotic stresses in plants. Members of the ERF family regulate stress responses mostly through the direct binding to specific promoter sequences (*cis*-acting GCC box) of defense-related genes^14^, but certain members have different binding sites. For instance, AtRAP2.2^15^ and AtRAP2.12^16^ belonging to ERF-VII subgroup in Arabidopsis, specifically recognize the motif ATCTA in the promoter of downstream genes. ERF genes have been identified in a number of plants, including Arabidopsis^17^, rice^18,19^, soybean^20^, and wheat (*Triticum aestivum*)^21^. Constitutive expression of soybean *GmERF3* in transgenic tobacco plants reduces susceptibility to high salinity, dehydration, fungal, and viral diseases^22^. Ectopic overexpression of *JERF1* and *JERF3* increases resistance to drought and osmotic stress in tobacco and rice^23,24^. Transgenic Arabidopsis plants with overexpressed *AtERF98* exhibit enhanced tolerance to salt via regulation of ascorbic acid synthesis^25^. Moreover, the biological role of ERFs in waterlogging stress has been reported previously. Overexpression of an ERF gene *Sub1A* leads to an enhanced tolerance to water submergence in rice^26–28^. Licausi et al.^29^ found that double mutants of *hre1* and *hre2*, two hypoxia-inducible *ERFs* in Arabidopsis, display increased sensitivity to anoxia. *HRE1* overexpression improves anoxia tolerance of transgenic Arabidopsis plants, and increases the PDC and ADH activities. Transgenic Arabidopsis plants constitutively expressing *BnERF2.4* from *Brassica napus* exhibit enhanced submergence tolerance and alleviated oxidative damage^30^. However, the molecular mechanism of how ERFs regulate waterlogging tolerance remains largely unknown.

Petunia, an important horticultural plant that is highly sensitive to submergence, is an excellent model system for studies of waterlogging responses. In previous work, we identified a cluster of transcription factors during petunia flower development via transcriptomic analysis, including some ERFs^31^. We have recently reported a critical role of *PhERF2* in antiviral RNA silencing and also observed that expression levels of *PhERF2* were significantly induced by stress-related hormones including ethylene, abscisic acid, salicylic acid, and methyl jasmonate as well as abiotic stress treatments such as cold, NaCl, and water stress^32^. The fact that roles of PhERF2 homologs in tolerance to abiotic stresses, such as salt^33^ and cold^34^, are characterized in other species prompts us to hypothesize that PhERF2 is involved in the stress regulation. Here, we report an additional function of *PhERF2* in petunia waterlogging tolerance. *PhERF2* silencing reduced petunia tolerance to waterlogging, and its overexpression increased the tolerance. Our results support an important role of *PhERF2* in the regulation of waterlogging resistance in petunia.

## Materials and methods

### Plant materials and growth conditions

Petunia (*Petunia* *×* *hybrida*) cultivar ‘Mitchell Diploid’ was used for generation of stable transformants. Seeds of wild-type (WT) and homozygous T2 generation transgenic petunia plants were surface-sterilized with 75% ethanol and 5% NaClO. After rinsing five times with sterilized water, WT and transgenic petunia seeds were placed on Murashige and Skoog (MS) medium without antibiotics or containing 50 mg L^−1^ kanamycin, respectively. Two weeks post germination, transgenic petunia seedlings were transferred to non-antibiotic MS medium to continue growth for 1 week. Both were then transplanted into the soil mixture and kept at 23/19 °C day/night under a photoperiod of 12/12 h light/dark. To determine transcript abundances of genes in petunia plants exposed to waterlogging stress, uppermost or lower leaves at different times after treatment were harvested for RNA extraction.

### Generation of transgenic petunia plants

A 339-bp fragment and a full length 1143-bp fragment harboring the ORF region of *PhERF2* were PCR-amplified and cloned into pGSA1285 and pGSA1403 vectors for generating the RNAi and overexpression constructs, respectively, as previously described^32^. The constructs were transformed into *Agrobacterium tumefaciens* strain LBA4404 (Takara, Otsu, Shiga, Japan) via electroporation. For the electroporation, 20 μl of LBA4404 competent cells were mixed with 0.1–0.2 μg of constructed plasmids and subjected to an electrical pulse at 2.5 kV and 400 Ω in a cold cuvette using a Gene Pulser (Bio-Rad, Richmond, CA, USA). The cells were suspended in 1 ml of liquid LB medium for 2 h of incubation at 28 °C, and then spread on solid LB medium containing appropriate antibiotics for selection of positive colonies. Leaf discs of petunia ‘Mitchell Diploid’ were used for inoculation with *Agrobacterium* according to previous description^35^. The resulting transformants were selected on MS plates supplemented with 100 mg L^−1^ kanamycin^36^. After a continuous cultivation, the *PhERF2*-RNAi (1A, 1B, and 4B) and *PhERF2*-overexpressing (C, D, and I) lines in the T2 generation exhibiting 100% survival on MS selection medium were obtained and used for further waterlogging assay.

### Waterlogging treatments

To examine the impact of waterlogging stress on WT, *PhERF2*-RNAi, and -overexpressing plants, the following treatments were applied to 5-week-old petunia plants before morphology was recorded: 0, 24 h waterlogging, 4 days waterlogging +14 days recovery. All treatments were replicated 5 times and 16 plants were used for each replication. Plants were subjected to 24 h continuous flooding treatment by putting small pots with plants into larger ones, which were then excessively irrigated with tap water at room temperature. For flooding experiments, the water level was kept at 2–3 cm higher than soil surface during the flooding process. After the treatment, a complete drainage was ensured through a drilled hole located underneath the pot. The plants untreated with flooding were used as a control. We hypothesize that transcriptional responses to waterlogging occur much earlier than physiological and metabolic processes and morphological alteration. Therefore, for expression analysis of *ADH*s, *PDC*s, and *LDH*, leaf samples with three biological replicates from 6-week-old WT and transgenic plants were collected at 12 h after waterlogging treatments.

### Measurement of chlorophyll content

Total chlorophyll (a + b) content was measured as described previously^37^. Briefly, fresh leaf samples (0.1 g) were extracted for 24 h with 10 ml of acetone:anhydrous ethanol (1:1, v/v) mixture solution in the dark at room temperature. The absorbance of resulting solvent was measured at both 663 and 645 nm using a Beckman Coulter DU 800 spectrophotometer (Beckman Coulter Inc., Fullerton, CA, USA). Chlorophyll contents were calculated based on the fresh weight.

### Cell death assay

The 20–30 mm of distal roots from WT and transgenic lines at various times after treatment with waterlogging were fixed by immersion in a mixture solution of formalin:ethanol:glacial acetic acid (18:1:1, v/v) at room temperature for at least 24 h under vacuum. After that, the samples were washed three times in 0.1 M phosphate buffer (pH 7.4) and dehydrated in a graded series of ethanol (70–100%), followed by subsequent air drying. A confocal laser-scanning microscope (LSM 700, Carl Zeiss, Germany) was used for examination of the cells. The histological detection of root nuclei was performed through staining with 1 mg L^−1^ DAPI (4', 6-diamidino-2-phenylindol dihydrochloride) in 10 mM Tris/HCl (pH 7.4), and then examining under a confocal laser-scanning microscope. Over 100 cells for each sample were observed for any DNA condensation and moon-shaped nuclei, an indication of PCD.

### Quantitative real-time PCR

Total RNA extraction was performed on petunia leaves using TRIzol reagent (Invitrogen, Carlsbad, CA, USA). The isolated RNA samples were purified with RNase-free DNase I (Promega, Madison, WI, USA), and subsequently transcribed to first-strand cDNA using oligo(dT)_20_ primer with SuperScript III reverse transcriptase (Invitrogen, Carlsbad, CA, USA), according to manufacturer’s instructions. Quantitative real-time PCR analysis was conducted using the SYBR Green PCR Master Mix (2×) (ABI7300; Applied Biosystems, Foster City, CA, USA) with cDNA being templates. Constitutively expressed *26S ribosomal RNA* was used as a reference gene to standardize cDNA^38,39^. Oligonucleotide primers used for detection of gene transcripts are shown in Supplementary Table S1.

### Electrophoretic mobility shift assay

Electrophoretic mobility shift assay (EMSA) was performed as previously described^40^ with minor changes. The complete coding region of *PhERF2* was amplified and cloned into the pET28a vector. The overexpression of the corresponding PhERF2 protein was achieved through the culturing of transformed *E. coli* Rosetta (DE3) cells by addition of the inducer isopropylthio-β-galactoside at 16 °C for 24 h. The recombinant proteins were extracted and applied to the column (HisTrap HP, GE) for purification according to the manufacturer’s instructions. The biotin-labeled WT or site-directed mutant probe containing a 35-bp oligonucleotide in the *ADH1-2* promoter was amplified using the primers Bio-pADH1-2-F/pADH1-2-R or Bio-mpADH1-2-F/mpADH1-2-R, respectively (Supplementary Table S1). The non-labeled probe served as the competitor. Subsequently, the interaction of PhERF2 with *ADH1-2* promoter was carried out using the LightShift EMSA Optimization and Control Kit (Pierce, Thermo Fisher Scientific, MA, USA). The protein-DNA complexes were separated by electrophoresis on a Tris-glycine-buffered 6% non-denaturing polyacrylamide gel. The binding signals of PhERF2 with biotin-labeled probe were detected using the Chemiluminescent Nucleic Acid Detection Module Kit (Pierce, Thermo Fisher Scientific, MA, USA).

### Dual luciferase assay

Dual luciferase assay was performed using a method previously described^41^. The full-length coding sequence of *PhERF2* was amplified and inserted into pGreenII62-SK vector as the effector, driven by CaMV 35S promoter, and the empty vector was used as a control. To generate the reporter construct, a 1256-bp *ADH1-2* promoter sequence bearing four putative binding sites of PhERF2 (Supplementary Fig. S1) was ligated into transient expression pGreenII0800-LUC vector, with the PhERF2 targeted candidate *ADH1-2* promoter driving a firefly luciferase (LUC) gene and a CaMV 35 promoter driving a *Renilla* luciferase (REN) gene. The primers used for these two constructs are listed in Supplementary Table S1. To examine the transactivation of PhERF2 to *ADH1-2* promoter, the *A. tumefaciens* GV3101 cells transformed with constructed effector and reporter plasmids were used to co-infiltrate 6-leaf-stage petunia seedlings. The enzyme activities of LUC and REN were detected using dual-luciferase assay kit (Promega, Madison, WI, USA) on a luminometer Tecan Infinite M200 (Männedorf, Switzerland). The promoter activities were indicated by the LUC/REN ratio.

### Statistical analysis

All experiments were carried out with a minimum of three biological replicates for different individual plants. The significance of difference was determined through Student’s *t* test at *P* value < 0.05, using JMP (Version 11.0) software (SAS Institute Inc., Cary, NC, USA).

## Results

### *PhERF2* affects tolerance of petunia to waterlogging

**Fig. 1:**
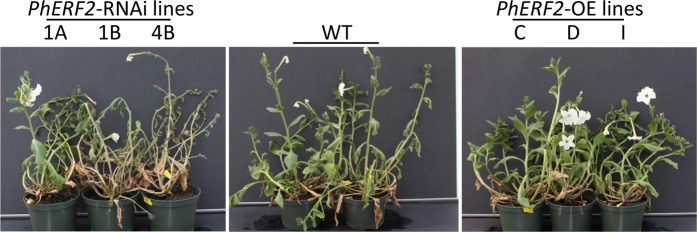
Representative phenotypes of *PhERF2*-silenced and -overexpressing seedlings exposed to waterlogging and recovery. Five-week-old seedlings of wild type (WT), *PhERF2*-RNAi (1A, 1B, and 4B), and *PhERF2*-overexpressing (OE) lines (C, D, and I) were subjected to flooding treatment for 4 days, and then recovery for 14 days. Photographs were taken at 14 days post recovery

**Fig. 2:**
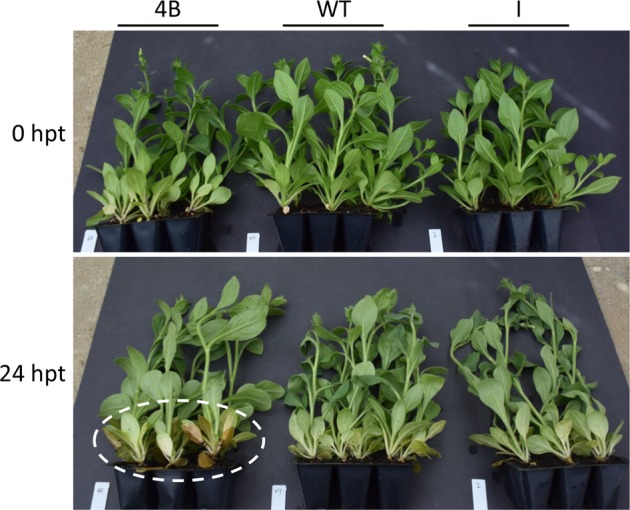
Representative phenotypes of *PhERF2*-silenced and -overexpressing seedlings following 24 h of waterlogging treatment. Five-week-old seedlings of wild type (WT), *PhERF2*-RNAi (4B), and *PhERF2*-overexpressing (I) lines were subjected to flooding treatment. Photographs were taken at 0 h and 24 h post treatment (hpt). The phenotype of leaf chlorosis is marked in a dashed circle

To investigate the role of *PhERF2* in waterlogging responses, transgenic petunia plants with RNAi silencing (lines 1A, 1B, and 4B) and overexpression (lines C, D, and I) of *PhERF2* were generated, respectively. Waterlogging treatment triggered wilting and leaf chlorosis in all tested plants of transgenic lines, but the symptoms occurred earlier and more seriously in *PhERF2*-RNAi lines than *PhERF2*-overexpressing and WT plants (Fig. 1). After waterlogging for 4 days and recovery for 14 days, the *PhERF2*-silenced plants were severely damaged and suffered 96% mortality, whereas nearly all *PhERF2*-overexpressing plants survived and displayed a quicker and stronger recovery than WT plants (Fig. 1). At 24 h post waterlogging, particularly, older leaves at the bottom of the plant became chlorotic and rotten in *PhERF2*-RNAi lines, while the leaves of WT and transgenic lines overexpressing *PhERF2* exhibited much milder symptoms with relatively healthy leaves, compared with the non-waterlogged controls (Fig. 2).

### Chlorophyll accumulates differently in WT and *PhERF2* transgenic plants under waterlogging

**Fig. 3:**
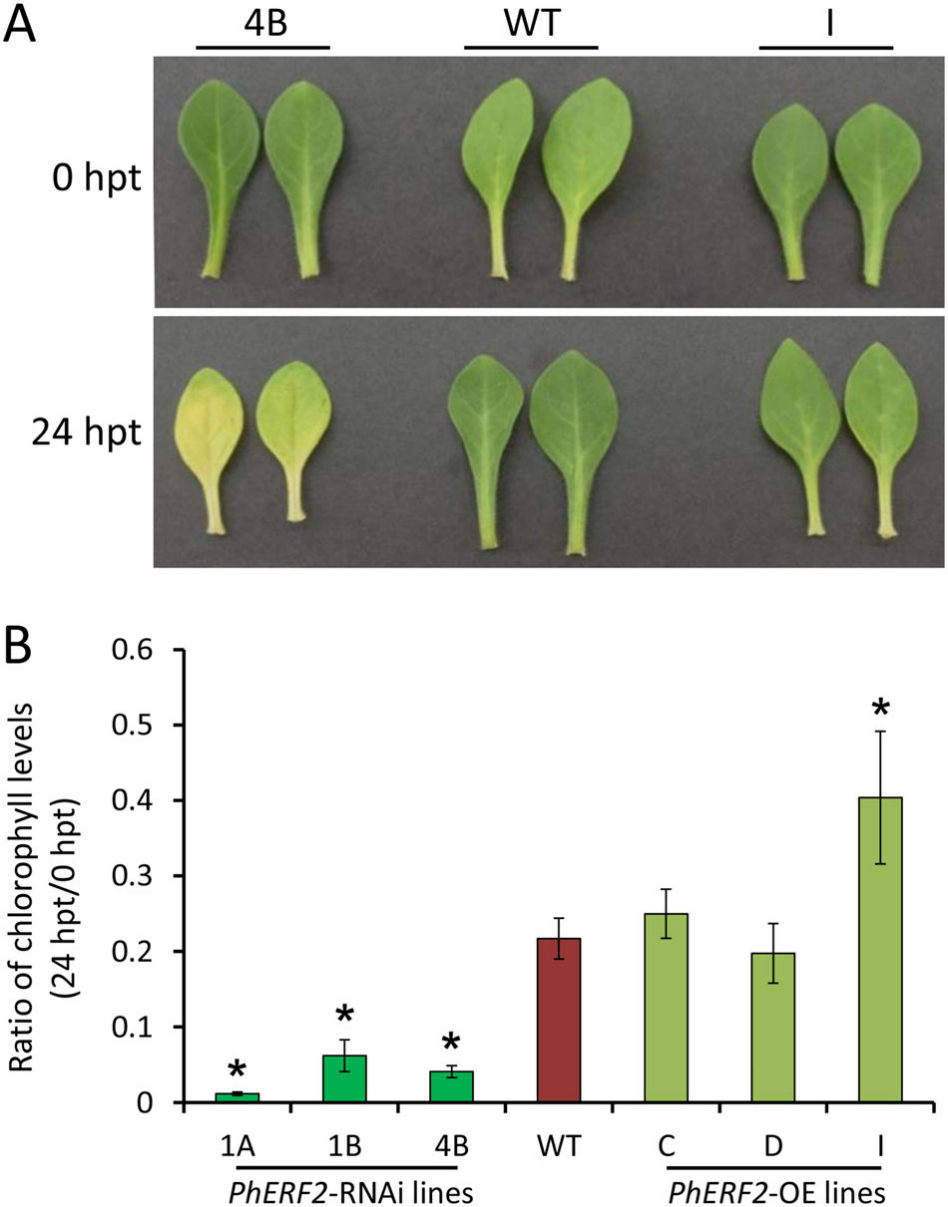
Chlorophyll levels of leaves from *PhERF2*-silenced and -overexpressing seedlings under waterlogging conditions. Representative phenotypes of leaves (**a**) and ratio of chlorophyll levels (**b**) of 5-week-old WT, *PhERF2*-RNAi, and *PhERF2*-overexpressing (OE) lines subjected to flooding treatment. The basal leaves photographed were harvested at the bottom of plants 0 h and 24 h post treatment (hpt), and the percentage of chlorophyll content was calculated at 24/0 hpt. Data represent the means (±SD) of three biological replicates. Asterisks denote statistical significance using Student’s *t* test at *P* < 0.05

After 24 h of exposure to waterlogging stress, the petunia WT, *PhERF2*-RNAi, and -overexpressing lines showed variable leaf chlorosis and yellowing symptoms (Figs. 2, 3a). To further determine the leaf symptoms change, we measured chlorophyll contents of older leaves at the bottom of the WT and transgenic plants. By comparison with control lines without waterlogging stress, the leaf chlorophyll levels of WT and *PhERF2* transgenic lines were decreased rapidly and significantly in response to waterlogging (Fig. 3b). The ratio of chlorophyll content at 24 h post treatment (hpt) to that at 0 hpt in WT plants was substantially higher than in *PhERF2*-silenced lines but lower than in *PhERF2*-overexpressing lines (Fig. 3b). Specifically, the chlorophyll content was decreased by 78.3% in WT plants, by 74.9%, 79.9%, and 59.6% in three overexpression lines (C, D, and I), and by 98.8%, 93.8%, and 95.9% in three silencing lines (1A, 1B, and 4B) at 24 h of waterlogging stress, respectively, compared to corresponding unstressed control.

### Waterlogging treatment induces *PhERF2* expression

**Fig. 4:**
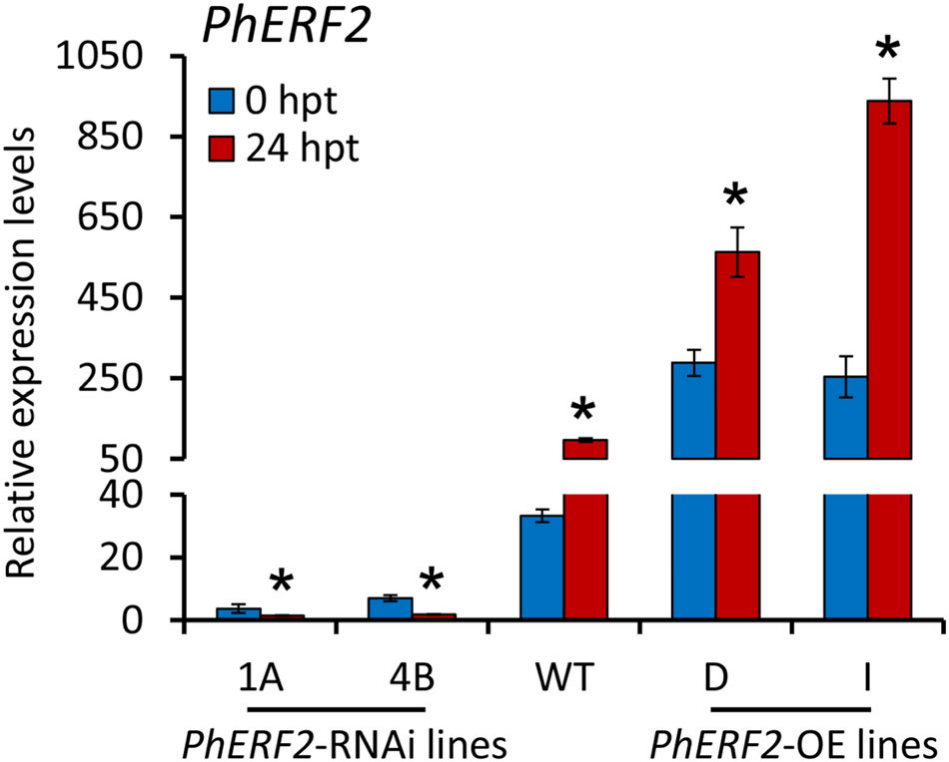
Expression of *PhERF2* in *PhERF2*-silenced and -overexpressing seedlings under waterlogging conditions. Quantitative real-time PCR analysis of *PhERF2* expression in the leaves of WT, *PhERF2*-RNAi lines (1A and 4B) and *PhERF2*-overexpressing (OE) lines (D and I) at 0 h and 24 h post treatment (hpt) with waterlogging. Uppermost leaves of 5-week-old plants were used. Data represent the means (±SD) of three biological replicates. Transcript abundances were standardized to *26S rRNA*. Asterisks denote statistical significance using Student’s *t* test at *P* < 0.05

To study the expression of *PhERF2* in response to waterlogging stress, 5-week-old plants of WT, *PhERF2*-overexpressing (D and I) and *PhERF2*-RNAi (1A and 4B) were waterlogged, and transcript levels of *PhERF2* in uppermost leaves were determined by quantitative real-time PCR. *PhERF2* expression levels were significantly higher in the overexpression lines and dramatically lower in the silenced lines than in the WT plants (Fig. 4). Transcripts of *PhERF2* were markedly up-regulated by waterlogging in the WT and overexpression lines, especially line D and I. A 2.1-fold and 3.7-fold increase of *PhERF2* expression in response to 24 h of waterlogging was observed for the overexpression lines, respectively. Two *PhERF2*-RNAi lines showed remarkable reduction in transcript abundance of *PhERF2* under waterlogging stress (Fig. 4).

### Overexpression of *PhERF2* induced PCD in response to waterlogging

**Fig. 5:**
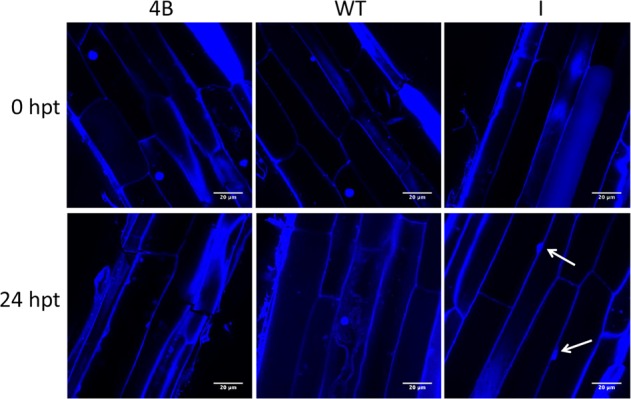
The programmed cell death in the roots of *PhERF2*-overexpressing lines subjected to waterlogging. DAPI staining was used to identify cell death at 0 h and 24 h post treatment (hpt) with waterlogging. Arrows denote the moon-shaped nuclei

To better understand the impact of *PhERF2* on waterlogging tolerance at the cellular level, we assessed the cell microstructures of roots from WT and transgenic plants untreated and treated with waterlogging. In the non-waterlogged roots, no cell death was observed, and all the cells and inner nuclei remained intact (Fig. 5). DAPI staining detected cell death in the roots of WT and *PhERF2*-RNAi (4B) plants after 24 h of waterlogging. Waterlogging caused a loss of cell integrity via lysis and extensive collapse of cell internal structures, with many cells losing protoplasms and generating distorted organelles (Fig. 5). In contrast, waterlogging stress resulted in DNA condensation and the formation of moon-shaped nuclei in waterlogging-tolerant *PhERF2*-overexpressing lines (I) (Fig. 5).

### *PhERF2* mediates alcoholic and lactate fermentations

**Fig. 6:**
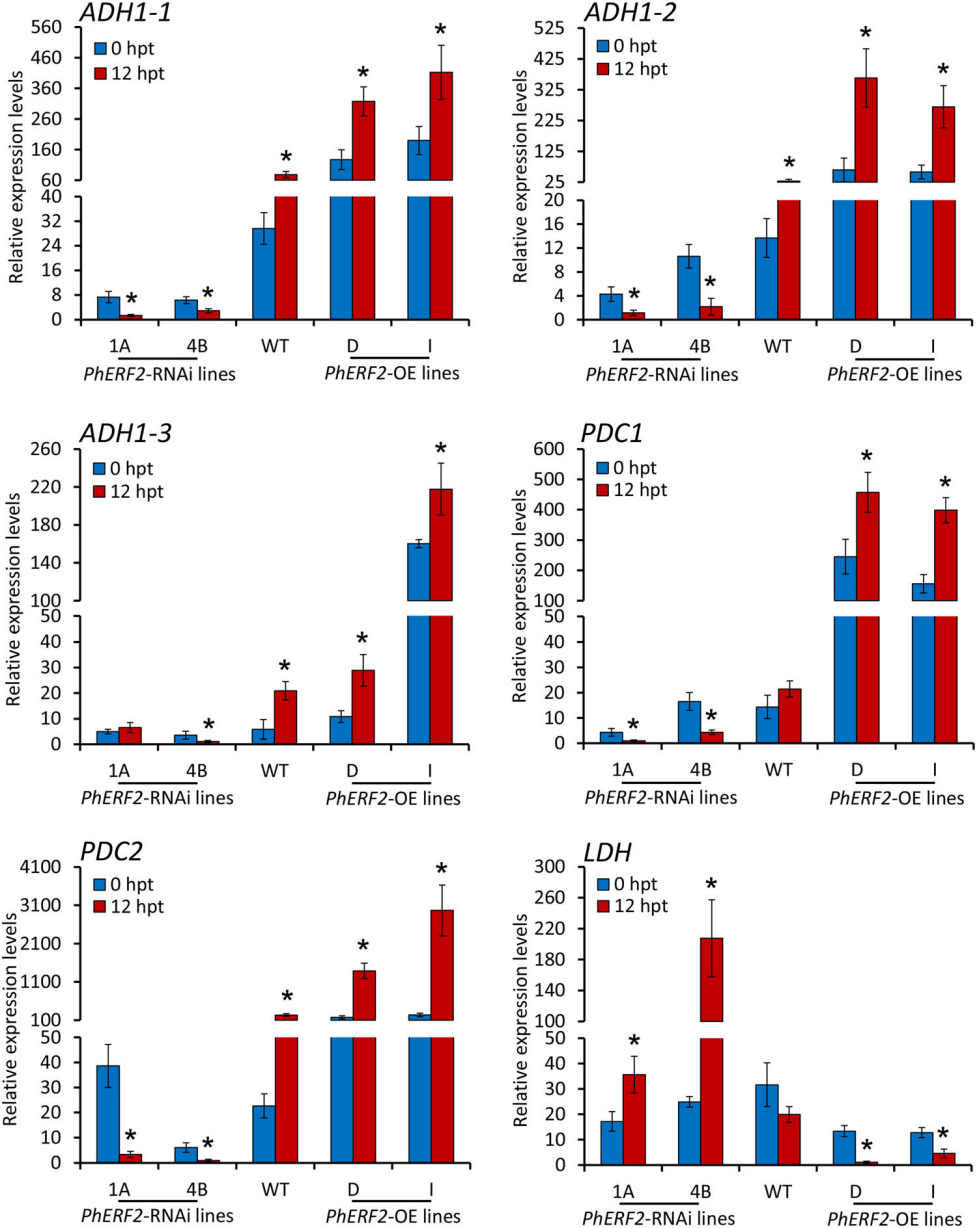
Expression of lactate and alcoholic fermentation-related genes in *PhERF2*-silenced and -overexpressing seedlings under waterlogging conditions. Quantitative real-time PCR analysis of transcript abundances for *ADH1-1*, *ADH1-2*, *ADH1-3*, *PDC1*, *PDC2*, and *LDH* in the leaves of WT, *PhERF2*-RNAi lines (1A and 4B) and *PhERF2*-overexpressing (OE) lines (D and I) at 0 h and 12 h post treatment (hpt) with waterlogging. The leaves at the bottom of 6-week-old plants were used for expression analysis. Data represent the means (±SD) of three biological replicates. *26S rRNA* was used as an internal control. Asterisks denote statistical significance using Student’s *t* test at *P* < 0.05

Hypoxia resulting from waterlogging initiates a switch from aerobic respiration to anaerobic fermentation via activation of the glycolytic and fermentation pathways. To study the correlation of *PhERF2* with fermentation pathways, transcript levels of a number of relevant genes, including three homologous genes to *ADH1*, two PDC genes, and one LDH gene, were examined. Compared to control plants without waterlogging, transcript abundances of the alcoholic fermentation-related genes *ADH1-1*, *ADH1-2*, *ADH1-3*, *PDC1*, and *PDC2* were decreased in *PhERF2*-silenced plants but increased in *PhERF2*-overexpressing plants under waterlogging treatment (Fig. 6). However, *PhERF2*-silenced plants showed increased transcript abundances of the lactate fermentation-related gene *LDH*, while *PhERF2*-overexpressing plants showed reduced *LDH* expression levels under waterlogging conditions. Thus, overexpression of *PhERF2* may activate the expression of alcoholic fermentation enzyme genes and alleviate the hypoxic conditions in the overexpression lines, protecting plants from waterlogging damage. The results suggest that a main pathway of NAD^+^ regeneration in waterlogged *PhERF2*-overexpressing plants is possibly not lactate fermentation but alcoholic fermentation. On the contrary, *PhERF2*-silenced plants are probably dependent on lactate fermentation against waterlogging stress.

### PhERF2 binds to the *ADH1-2* promoter

**Fig. 7:**
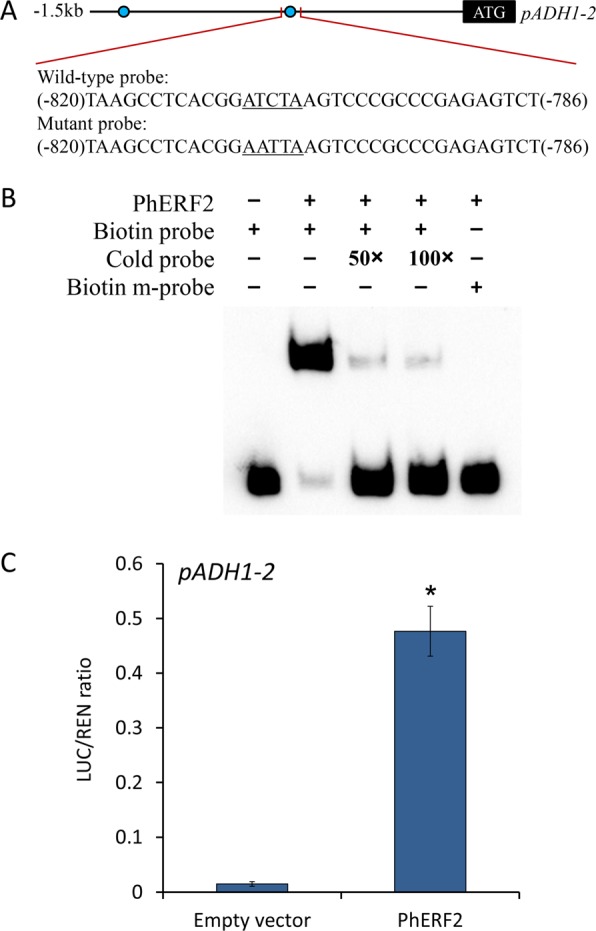
Transactivation of the *ADH1-2* promoter by PhERF2. **a** Graphic representation of petunia *ADH1-2* promoter (*pADH1-2*) with a 1.5 kb region upstream of the coding sequence. Two putative PhERF2 binding sites (ATCTA) are marked by blue circles. The probe sequences used for electrophoretic mobility shift assay (EMSA) are indicated, with the wild-type *cis*-element and its nucleotide substitutions being underlined, respectively. **b** EMSA of PhERF2 binding to the biotin-labeled probe. Non-labeled probes (cold) at 50- and 100-fold concentrations were added for competition, and mutant probe (m-probe) for binding specificity test. **c** Dual luciferase transient expression assay of the *ADH1-2* promoter (*pADH1-2*). The binding activity was expressed as a ratio of LUC to REN. LUC, firefly luciferase; REN, *Renilla* luciferase. Error bars represent the standard error of the means from three biological replicates. Statistical significance was determined using Student’s *t* test (*P* < 0.05) and denoted by asterisks

To further investigate the regulatory mechanism of PhERF2, we searched the promoter regions of those genes with significantly changed expression in transgenic lines. Based on the previously reported binding sites of AtRAP2.12^16^, an ortholog of PhERF2, we found four motifs with the core ATCTA (or TAGAT in the opposite strand) element in the 1.5 kb promoter region upstream of the *ADH1-2* coding sequence (Supplementary Fig. S1). A 35-bp fragment spanning positions −786 to −820 of the *ADH1-2* promoter was used as a probe for EMSA experiments (Fig. 7a). A clear binding of PhERF2 protein to the biotin-labeled target probe was visualized as slowed bands in the polyacrylamide gel, whereas no signals were detected from the protein-mutant probe (m-probe) complex (Fig. 7b). Additionally, a dual luciferase transient expression assay based on the effector and reporter constructs (Supplementary Fig. S2) was conducted to test whether the transactivation of *ADH1-2* promoter by PhERF2 occurred in petunia plants. Compared with empty vector control, the co-expression of *35S*::*PhERF2* and *pADH1-2*::*LUC* resulted in a 32-fold increase in LUC activity (Fig. 7c). The results revealed the direct interaction between PhERF2 and *ADH1-2* promoter.

## Discussion

In this study, we analyzed the morphological and physiological variations between WT and transgenic petunia plants, *PhERF2*-overexpressing and *PhERF2*-RNAi lines, under waterlogging conditions. There were no significant differences in morphology of plants without waterlogging treatment (Figs. 2, 3). After 24 h of waterlogging treatment, the leaves of the *PhERF2*-RNAi lines became yellow and withered. The results were in accordance with those reported previously about waterlogging effects on chrysanthemum^42^.

The waterlogging treatment resulted in reduced respiration and photosynthesis of plants due to lack of oxygen^43^. The plants recovered once the waterlogging ceased. After a successive waterlogging and recovery treatment, the *PhERF2*-RNAi plants were almost dead with all the leaves being severely wilted (Fig. 1), whereas the *PhERF2*-overexpressing plants were still alive with normal growth, suggesting that they probably used the *PhERF2*-regulated pathways to recover from the damage. Our data demonstrated that PhERF2 functions as a positive regulator in plant defense against waterlogging stress. Despite the recovery time of up to 14 days, in contrast to overexpression lines, the WT plants failed to completely recover and showed leaf chlorosis and wilting symptoms. It is quite likely that the plants may suffer some damage during the recovery period after waterlogging stress in this experiment. The physiological changes of WT and *PhERF2* transgenic plants during the transition phase from waterlogging to recovery require further examination.

Under waterlogging conditions, the levels of leaf chlorophyll decline significantly in plants^10,44^. For example, waterlogging results in reduced leaf chlorophyll content in both waterlogging-tolerant and -susceptible plants of pigeon pea (*Cajanus cajan*)^45^. In our study, the chlorophyll content also declined in both WT and transgenic lines under waterlogging conditions, but the chlorophyll levels were higher in *PhERF2*-overexpressing lines than in *PhERF2*-RNAi lines (Fig. 3). Plants treated with flooding suffer from hypoxia and chlorophyll degradation^2^. It appears that The *PhERF2*-RNAi lines suffered more from waterlogging stress than WT or overexpression lines. It seems likely that the chlorosis in the older leaves at the bottom of the plant is attributed to the translocation of nitrogen from lower, older to upper younger leaves under threat from waterlogging^46^.

Expression analysis indicated that *PhERF2* was significantly up-regulated in WT and overexpression plants under flooding conditions (Fig. 4). The *PhERF2* homologs in other species have similar expression profiles after treatments with abiotic stresses. Expression levels of *NtCEF1* from tobacco are elevated under cold and salt conditions^47^. Salt treatment increases transcript abundances of *JERF1*^33^ and *JERF3*^48^ in tobacco, and transgenic plants overexpressing these two genes showed decreased susceptibility to salt stress. Pepper *CaPF1* expression is markedly induced by low temperature and salt treatments, and ectopic expression of this gene confers tolerance to freezing in Arabidopsis^34^. Besides waterlogging tolerance, thus, a possible role of *PhERF2* in plant tolerance to various abiotic stresses should be further examined in the future. Furthermore, up-regulation of *PhERF2* under waterlogging suggested a critical involvement of *PhERF2* in plant against hypoxic stress and further recovery from waterlogging damage.

Aerenchyma formation occurs following programmed cell death (PCD), which facilitates oxygen capture of waterlogged tissues^49^, eases the hypoxic stresses, and improves the recovery and maintenance of aerobic respiration in plants under waterlogging^50,51^. The occurrence of PCD under waterlogging raises the survival chances of many plant species^52–54^. At the morpho-anatomical level, the responses of *PhERF2*-overexpressing plants to hypoxia were observed with induced PCD, probably leading to aerenchyma development in the roots (Fig. 5). It represents a positive adaptation to waterlogging^55^, since aerenchyma is beneficial to capture oxygen and to store and exchange gases within the waterlogged parts of plants^50^. However, *PhERF2*-RNAi plants may not be able to respond in any of these ways due to cell damage, thereby failing to survive under waterlogging stress. It seems likely that *PhERF2* is involved in the transcriptional regulation of PCD, generally acting as a precursor for aerenchyma formation in plants exposed to waterlogging.

The waterlogging stress affects diverse physiological and metabolic processes in plants^56,57^. One major pathway to be affected is glycolytic fermentation, particularly manifesting as an increase in alcohol fermentation with involvement of two key enzymes, PDC and ADH^9^. Induction of LDH-catalyzed lactate fermentation has also been demonstrated in some plant species at the initial stage of hypoxia^58^. Although *PhERF2*-overexpressing plants responded during waterlogging with an elevation in transcript abundances of *PDC* and *ADH*, *LDH* was not up-regulated but down-regulated (Fig. 6). This indicates that lactate fermentation rather than alcohol fermentation predominantly contributes to NAD^+^ regeneration in waterlogged *PhERF2*-overexpressing plants, while waterlogged *PhERF2*-silenced plants rely on lactate fermentation, which is a typical feature of plants sensitive to hypoxia^59^. This is consistent with our previous results in the waterlogging-susceptible chrysanthemum cultivar ‘13-13’^60^ and *Dendranthema nankingense* (Nakai) Tzvel^37^. Therefore, activation of alcoholic fermentation was considered as one effective strategy for plants to survive under strict anaerobiosis, as demonstrated in the seedlings of cucumber^61^ and chrysanthemum^62^ upon exposure to waterlogging stress.

ERFs represent a large family of transcription factors implicated in diverse biological processes, such as plant growth, development, and stress responses^63^. In Arabidopsis, approximately 122 putative ERF genes with conserved AP2/ERF domains are found in its genomic DNA^64^. Although many stress-related members are known to bind GCC box elements sharing a core AGCCGCC motif^65^, the binding specificity for various ERFs transcription factors appears to be very complicated. Here, our data revealed the specific activation of *ADH1-2* promoter containing ATCTA motif by PhERF2 (Fig. 7), which is consistent with previous reports on the binding activity of AtRAP2.12^16^, a homolog of PhERF2.

**Fig. 8:**
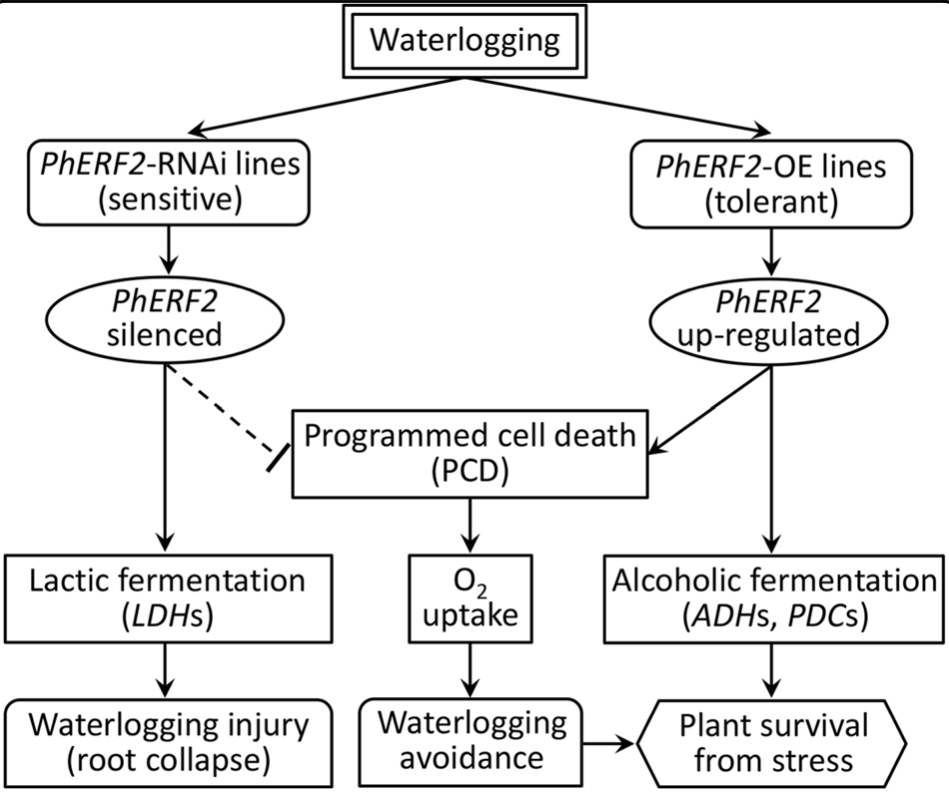
A proposed model for PhERF2’s function in waterlogging responses. PhERF2 positively regulates the programmed cell death and genes associated with the adaptation to waterlogging stress. OE, overexpressing; PCD, programmed cell death; LDH, lactate dehydrogenase; ADH, alcohol dehydrogenase gene; PDC, pyruvate decarboxylase gene. Positive regulation ; Negative regulation

Survival of plants under waterlogging stress is associated with avoidance/escape and endurance^66^. Petioles of submerged *Rumex palustris* plants elongate to allow the leaf lamina to protrude from the water thus restoring normal respiration^67^. Similarly, internode elongation occurs to avoid waterlogging stress in submerged rice^68^. This escape response is modulated by the endogenous hormone ethylene, an important signal in response to submergence^62^. For lowland rice, the waterlogging response involves endurance rather than escape^66^. In this work, we proposed a model of *PhERF2*’s role in tolerance of petunia to waterlogging (Fig. 8). *PhERF2* expression was increased in WT and *PhERF2*-overexpressing lines after waterlogging. Modulation of *PhERF2* expression affected the expression of alcoholic or lactate fermentation-related genes. As a less efficient energy supplier than aerobic respiration, anaerobic fermentation produces the toxic byproducts ethanol and acetaldehyde, leading to cellular metabolism disturbance and root collapse. Thus the timing of the shift from alcoholic to lactic fermentation could be an essential indicator of plant survivability under hypoxia and reduce damage^69^.

It is worth mentioning that *PhERF2* may participate in the formation of lysigenous aerenchyma through PCD in the roots of overexpression lines. This tissue is responsible for oxygen supplies to submerged roots and therefore alleviates hypoxia. This kind of avoidance strategy is widely employed in plants^49,52,66,70,71^. By comparison, the silenced lines without PCD appear to activate the lactic fermentation but not alcoholic fermentation pathway. Although this brings about temporary alleviation of energy shortage, it is insufficient to satisfy the ATP demand and simultaneously produces byproducts lactic acid disturbing cellular metabolism^13^. Thus in the waterlogging-susceptible *PhERF2*-RNAi lines, excessive accumulation of these byproducts might cause root collapse ultimately.

Our results provide new evidence that PhERF2 transcriptionally regulates the PCD and genes of alcoholic fermentation system to protect plants from anaerobic respiration damage, and therefore plays an important role in defense responses against waterlogging stress. EMSA and dual luciferase assays confirmed the direct binding of PhERF2 to *ADH1-2* promoter in petunia. However, the mechanism on how PhERF2 and its target gene synergistically modulate plant defense against waterlogging is still unclear. To further dissect the biological function of *PhERF2* in response to waterlogging, future studies should include comprehensive transcriptomic or metabolomic analyses in *PhERF2* transgenic plants.

## Supplementary information

Supplementary figure S1, S2, and table S1

## References

[1] IPCC. Climate Change 2007: Contribution of working group I to the fourth assessment report of the intergovernmental panel on climate change. (Cambridge University Press, Cambridge, 2007).

[2] Jackson M, Colmer T (2005). Response and adaptation by plants to flooding stress. Ann. Bot..

[3] Dennis ES (2000). Molecular strategies for improving waterlogging tolerance in plants. J. Exp. Bot..

[4] Purnobasuki H, Suzuki M (2004). Aerenchyma formation and porosity in root of a mangrove plant, *Sonneratia alba* (Lythraceae). J. Plant Res..

[5] Pennell RI, Lamb C (1997). Programmed cell death in plants. Plant Cell.

[6] Ismail AM, Ella ES, Vergara GV, Mackill DJ (2009). Mechanisms associated with tolerance to flooding during germination and early seedling growth in rice (*Oryza sativa*). Ann. Bot..

[7] James CS (2016). Does stream flow structure woody riparian vegetation in subtropical catchments? *Ecol*. Evol.

[8] Capon SJ, James CS, Williams L, Quinn GP (2009). Responses to flooding and drying in seedlings of a common Australian desert floodplain shrub: *Muehlenbeckia florulenta* Meisn. (tangled lignum). Environ. Exp. Bot..

[9] Kato-Noguchi H, Morokuma M (2007). Ethanolic fermentation and anoxia tolerance in four rice cultivars. J. Plant Physiol..

[10] Kumutha D, Sairam RK, Ezhilmathi K, Chinnusamy V, Meena RC (2008). Effect of waterlogging on carbohydrate metabolism in pigeon pea (*Cajanus cajan* L.): Upregulation of sucrose synthase and alcohol dehydrogenase. Plant Sci..

[11] Ismond KP, Dolferus R, de Pauw M, Dennis ES, Good AG (2003). Enhanced low oxygen survival in Arabidopsis through increased metabolic flux in the fermentative pathway. Plant Physiol..

[12] Maricle BR, Crosier JJ, Bussiere BC, Lee RW (2006). Respiratory enzyme activities correlate with anoxia tolerance in salt marsh grasses. J. Exp. Mar. Biol. Ecol..

[13] Vodnik D, Strajnar P, Jemc S, Mačeka I (2009). Respiratory potential of maize (*Zea mays* L.) roots exposed to hypoxia. Environ. Exp. Bot..

[14] Ohmetakagi M, Shinshi H (1995). Ethylene-inducible DNA binding proteins that interact with an ethylene-responsive element. Plant Cell.

[15] Welsch R, Maass D, Voegel T, DellaPenna D, Beyer P (2007). Transcription factor RAP2.2 and its interacting partner SINAT2: stable elements in the carotenogenesis of Arabidopsis leaves. Plant Physiol..

[16] Francesco L (2011). Oxygen sensing in plants is mediated by an N-end rule pathway for protein destabilization. Nature.

[17] Oñate-Sánchez L, Singh KB (2002). Identification of Arabidopsis ethylene-responsive element binding factors with distinct induction kinetics after pathogen infection. Plant Physiol..

[18] Cao Y, Song F, Goodman RM, Zheng Z (2006). Molecular characterization of four rice genes encoding ethylene-responsive transcriptional factors and their expressions in response to biotic and abiotic stress. J. Plant Physiol..

[19] Sharoni AM (2011). Gene structures, classification and expression models of the AP2/EREBP transcription factor family in rice. Plant Cell Physiol..

[20] Zhang G (2008). Phylogeny, gene structures, and expression patterns of the ERF gene family in soybean (*Glycine max* L.). J. Exp. Bot..

[21] Zhuang J (2011). Discovery and expression profile analysis of AP2/ERF family genes from *Triticum aestivum*. Mol. Biol. Rep..

[22] Zhang G (2009). Overexpression of the soybean *GmERF3* gene, an AP2/ERF type transcription factor for increased tolerances to salt, drought, and diseases in transgenic tobacco. J. Exp. Bot..

[23] Zhang H (2010). Functional analyses of ethylene response factor JERF3 with the aim of improving tolerance to drought and osmotic stress in transgenic rice. Transgenic Res..

[24] Zhang Z, Li F, Li D, Zhang H, Huang R (2010). Expression of ethylene response factor *JERF1* in rice improves tolerance to drought. Planta.

[25] Zhang Z, Wang J, Zhang R, Huang R (2012). The ethylene response factor AtERF98 enhances tolerance to salt through the transcriptional activation of ascorbic acid synthesis in Arabidopsis. Plant J..

[26] Xu K (2006). Sub1A is an ethylene-response-factor-like gene that confers submergence tolerance to rice. Nature.

[27] Fukao T, Xu K, Ronald PC, Bailey-Serres J (2006). A variable cluster of ethylene response factor-like genes regulates metabolic and developmental acclimation responses to submergence in rice. Plant Cell.

[28] Fukao T, Baileyserres J (2008). Submergence tolerance conferred by Sub1A is mediated by SLR1 and SLRL1 restriction of gibberellin responses in rice. Proc. Natl Acad. Sci. USA.

[29] Licausi F (2010). HRE1 and HRE2, two hypoxia-inducible ethylene response factors, affect anaerobic responses in *Arabidopsis thaliana*. Plant J..

[30] Lv Y, Fu S, Chen S, Zhang W, Qi C (2016). Ethylene response factor BnERF2-like (ERF2.4) from *Brassica napus* L. enhances submergence tolerance and alleviates oxidative damage caused by submergence in *Arabidopsis thaliana*. Plant J..

[31] Wang H (2013). Transcriptome changes associated with delayed flower senescence on transgenic petunia by inducing expression of *etr1-1*, a mutant ethylene receptor. PLoS ONE.

[32] Sun D (2016). A petunia ethylene-responsive element binding factor, *PhERF2*, plays an important role in antiviral RNA silencing. J. Exp. Bot..

[33] Zhang H (2004). The ethylene-, jasmonate-, abscisic acid- and NaCl-responsive tomato transcription factor JERF1 modulates expression of GCC box-containing genes and salt tolerance in tobacco. Planta.

[34] Yi SY (2004). The pepper transcription factor CaPF1 confers pathogen and freezing tolerance in Arabidopsis. Plant Physiol..

[35] Liang YC, Reid MS, Jiang CZ (2012). Controlling plant architecture by manipulation of gibberellic acid signalling in petunia. Hortic. Res.

[36] Yin J (2015). A basic helix-loop-helix transcription factor, PhFBH4, regulates flower senescence by modulating ethylene biosynthesis pathway in petunia. Hortic. Res..

[37] Yin DM, Chen SM, Chen F, Guan Z, Fang W (2010). Morpho-anatomical and physiological responses of two *Dendranthema* species to waterlogging. Environ. Exp. Bot..

[38] Chen JC (2004). Chalcone synthase as a reporter in virus-induced gene silencing studies of flower senescence. Plant Mol. Biol..

[39] Reid, M. S., Chen, J. C. & Jiang, C. Z. in *Petunia*. (eds Gerats, T. & Strommer, J.) 381–394 (Springer, Berlin Heidelberg, 2009).

[40] Liu P, Wu Z, Xue H, Zhao X (2017). Antibiotics trigger initiation of SCC*mec* transfer by inducing SOS responses. Nucleic Acids Res.

[41] Chen K, Liu H, Lou Q, Liu Y (2017). Ectopic expression of the grape hyacinth (*Muscari armeniacum*) R2R3-MYB transcription factor gene, *MaAN2*, induces anthocyanin accumulation in tobacco. Front. Plant Sci..

[42] Yin D, Zhang Z, Luo H (2012). Anatomical responses to waterlogging in *Chrysanthemum zawadskii*. Sci. Hortic..

[43] Ashraf MA (2012). Waterlogging stress in plants: a review. Afr. J. Agr. Res..

[44] Ahmed S, Nawata E, Hosokawa M, Domae Y, Sakuratani T (2002). Alterations in photosynthesis and some antioxidant enzymatic activities of mungbean subjected to waterlogging. Plant Sci..

[45] Kumutha D (2009). Waterlogging induced oxidative stress and antioxidant activity in pigeonpea genotypes. Biol. Plant..

[46] Drew MC, Sisworo EJ (1977). Early effects of flooding on nitrogen deficiency and leaf chlorosis in barley. New Phytol..

[47] Lee JH (2005). Functional characterization of NtCEF1, an AP2/EREBP-type transcriptional activator highly expressed in tobacco callus. Planta.

[48] Wang H (2004). Ectopic overexpression of tomato *JERF3* in tobacco activates downstream gene expression and enhances salt tolerance. Plant Mol. Biol..

[49] Suralta RR, Yamauchi A (2008). Root growth, aerenchyma development, and oxygen transport in rice genotypes subjected to drought and waterlogging. Environ. Exp. Bot..

[50] Colmer TD (2003). Long-distance transport of gases in plants: a perspective on internal aeration and radial oxygen loss from roots. Plant Cell Environ..

[51] Finlayson C (2005). Plant ecology of Australia’s tropical floodplain wetlands: a review. Ann. Bot..

[52] Voesenek LA, Colmer TD, Pierik R, Millenaar FF, Peeters AJ (2006). How plants cope with complete submergence. New Phytol..

[53] Ashraf M (2003). Relationships between leaf gas exchange characteristics and growth of differently adapted populations of Blue panicgrass (*Panicum antidotale* Retz.) under salinity or waterlogging. Plant Sci..

[54] Armstrong W (1987). The anatomical characteristics of roots and plant response to soil flooding. New Phytol..

[55] Wang W, Xiao Y, Chen L, Lin P (2007). Leaf anatomical responses to periodical waterlogging in simulated semidiurnal tides in mangrove *Bruguiera gymnorrhiza* seedlings. Aquat. Bot..

[56] Das KK, Panda D, Sarkar RK, Reddy JN, Ismail AM (2009). Submergence tolerance in relation to variable floodwater conditions in rice. Environ. Exp. Bot..

[57] Rich SM, Ludwig M, Colmer TD (2008). Photosynthesis in aquatic adventitious roots of the halophytic stem-succulent *Tecticornia pergranulata* (formerly *Halosarcia pergranulata*). Plant Cell Environ..

[58] Rivoal J, Hanson AD (1994). Metabolic control of anaerobic glycolysis, overexpression of *Lactate Dehydrogenase* in transgenic tomato roots supports the davies-roberts hypothesis and points to a critical role for lactate secretion. Plant Physiol..

[59] Nada K, El-Mowafy O (2011). Effect of precuring warming on mechanical properties of restorative composites. Int. J. Dent..

[60] Yin D, Chen S, Chen F, Guan Z, Fang W (2009). Morphological and physiological responses of two chrysanthemum cultivars differing in their tolerance to waterlogging. Environ. Exp. Bot..

[61] Kang YY, Guo SR, Li J, Duan J (2009). Effect of root applied 24-epibrassinolide on carbohydrate status and fermentative enzyme activities in cucumber (*Cucumis sativus* L.) seedlings under hypoxia. Plant Growth Regul..

[62] Yin D, Chen S, Chen F, Jiang J (2013). Ethylene promotes induction of aerenchyma formation and ethanolic fermentation in waterlogged roots of *Dendranthema* spp. Mol. Biol. Rep..

[63] Xu ZS, Chen M, Li LC, Ma YZ (2008). Functions of the ERF transcription factor family in plants. Bot.-Bot..

[64] Nakano T, Suzuki K, Fujimura T, Shinshi H (2006). Genome-wide analysis of the ERF gene family in Arabidopsis and rice. Plant Physiol..

[65] Hao D, Ohme-Takagi M, Sarai A (1998). Unique mode of GCC box recognition by the DNA-binding domain of ethylene-responsive element-binding factor (ERF domain) in plant. J. Biol. Chem..

[66] Hinz M (2010). Arabidopsis RAP2.2: an ethylene response transcription factor that is important for hypoxia survival. Plant Physiol..

[67] Peeters AJ (2002). Submergence research using *Rumex palustris* as a model; looking back and going forward. J. Exp. Bot..

[68] Hattori Y (2009). The ethylene response factors SNORKEL1 and SNORKEL2 allow rice to adapt to deep water. Nature.

[69] Mustroph A (2006). Organ-specific analysis of the anaerobic primary metabolism in rice and wheat seedlings. I: Dark ethanol production is dominated by the shoots. Planta.

[70] Gunawardena A, Pearce DM, Jackson MB, Hawes CR, Evans DE (2001). Characterisation of programmed cell death during aerenchyma formation induced by ethylene or hypoxia in roots of maize (*Zea mays* L.). Planta.

[71] Perata P, Voesenek L (2007). Submergence tolerance in rice requires *Sub1A*, an ethylene-response-factor-like gene. Trends Plant Sci..

