# Supplementary material for: PhERF2, an ethylene-responsive element binding factor, plays an essential role in waterlogging tolerance of petunia
Source: Hortic Res. 2019 Jul 1;6:83. doi: 10.1038/s41438-019-0165-z (PMC6804856; doi:10.1038/s41438-019-0165-z)
Supplement: Supplementary file 1 — Supplementary figure S1, S2, and table S1 [file 41438_2019_165_MOESM1_ESM.docx]

**Fig. S1 The promoter sequence upstream of petunia *ADH1-2* coding region.**

The putative binding motifs of PhERF2 were underlined in red. Bold types in square indicate the start codon of translation.

AGACGTGGGGGCGCAAGTCCCATGGATCTTATTTTTTAAGTATTTTAAATTAATAAAATAGCATAATGAGCCAAAAAAATATCAAAAGTACATAAAAAATTAAAAATAAGAAAAAAAATTATAGAAACAAAAAATCTAACATTTTTAACAATGGTAAGAAATTGAATAATATTACAGTTTTTGTGAGATACAAATAAACCATAAGGCCTTAACTTTTAAACTTAAAAATAAAAATTTGTTACTATAAAAAGTAAATAAACAACAAATATTTATTACTATTATAATTTAAAATATTAATTCTTCATTGATAAATATAAAATGGCTCTAAATTGTAAAATAATTGAAGTCTAACAATTTTGAGTGACGGAAATAAAACATGAAGACCAATAACAAGTGATGATGTTATAAACATCGGTTATGTTTTTTTTAGATTAAGTATACAGATGATATTCATCCTTATGATATTCTCAATTTATAATGCATGTAATATTATGTCTCGTTAATTAAGCTATACTCAATTTTTAAATTTCTATAGATGAATATAACTTCATTTTTAAAGAAATTGTGGATGAACAATCTTAATTAGGGAATTGGACAGATCATTTGCATAGGTTCTATAAGGCGAGTTCCGAGCGTTGGGCGTTGGGCGTGTCTGGGCGCATCGTCGGATGCTTGAGGCGTAAGCCTCACGGATCTAAGTCCCGCCCGAGAGTCTTAGGGCATTTTGCTAGTGCCCCGCGAATTCCGGGCGAGTCCCAAAACTGCCTTTTAAAACTGAGTATAAGACACGTCCGCTTAATCAAATTCAGTTTAAGGATTGAGGTTGGACTCTATTTTAAGCCTAACCTCAGTCACTCGGGACTCAAGTTGACGTCTCAAGGCTATTTGTTTGTATGAGTTGGCCTCAACTTCATGTCCGTGAACTACAGTTTTGTGGCTACAATTGCTAAATTATTTAGAAATTGACTACACCTTAAATGACAATGCTTTTCCAGACAGCAGCTAATTCTTAGGTAAATCATTTAATCAAATCTTTTGTGCATAATTAGAACACTTTCAAGTGATTTTTGAGCCTTTCACTTGAAAAATGAACTTAAAAATTATGAATTTTCCAGTTTTGAATAAAATTTGCTGTATTTTCTCGAGTGCCGAAAGAAAAAGAAAAACGAAGTCAAAACAATATTCCTGGGTCAAAGCATCCTTAAGGCTAAAGAAATCACATAATTAAACGTATCACTTTCTTGACAAAGACTCGAGGAGTTGACACAAAAACAAACAGCCAAAAACTGGTTTTGTAAACCAAGTGACTAAAACCTGCAAACGGTTTTACCATGGCCTATTGTTTTTTGTTTTTAATCCAAATTTCAGGTATAAATACCCCCAGCTAATGCTGTGTCTCATCACACTCAAGCAATTCAACTCAGTATTCAGAAACAAACAAAGGTCTCTGCAATTTATAAAGTTTTCATATAAGTACATTTTAGTGATCAGTAAAAGAAA**ATG**

**Fig. S2 Schematic diagrams of the effector and reporter constructs for dual luciferase assay.**

**
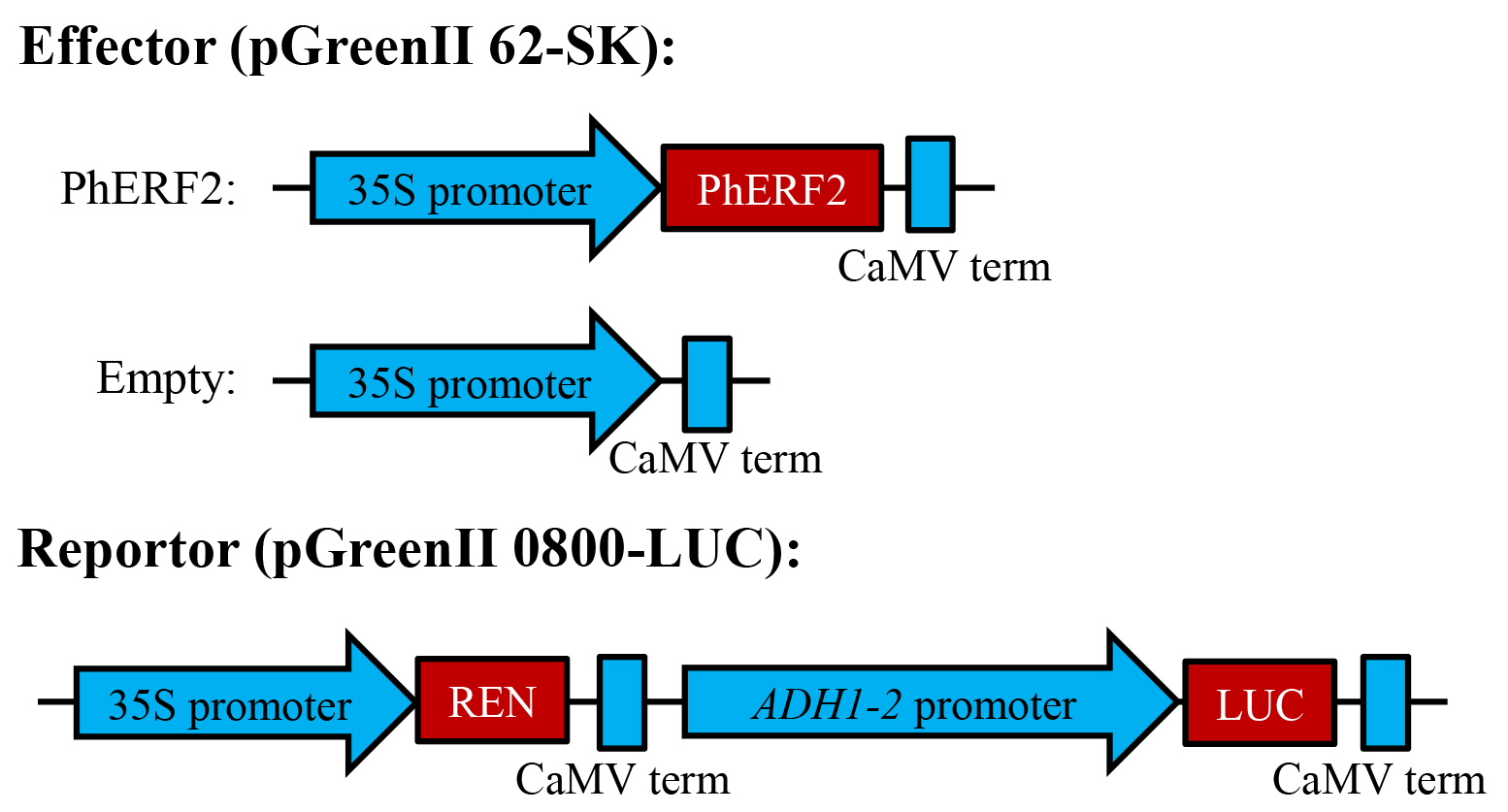
**

**Table S1 Primers used for quantitative real-time PCR, probe synthesis, and plasmid construct.**

| Primer name | Primer sequence (5’-3’) | Product size | Purpose |
| --- | --- | --- | --- |
| PhERF2-F | GGTGGTGCTATAATTTCCGATTA | 291bp | Quantitative real-time PCR for expression analysis |
| PhERF2-R | TCTCCGAAAATGGAACTTGG |  |  |
| PhADH1-1-F | TGCCATGATCTCTGCTTTTG | 245bp |  |
| PhADH1-1-R | TCTCCGAAAATGGAACTTGG |  |  |
| PhADH1-2-F | GCCTCGTTCTGATCTTCCTTC | 204bp |  |
| PhADH1-2-R | CAAGGAGCAGCGACTAGGAG |  |  |
| PhADH1-3-F | TCTTCGCTGCATCATCCATA | 195bp |  |
| PhADH1-3-R | CCTTGTAAGCGGCACAACTT |  |  |
| PhPDC1-F | TCAGAGGATGGTGTCTGCTG | 247bp |  |
| PhPDC1-R | GCATCGCAGCATTGTAGAAA |  |  |
| PhPDC2-F | GTGAAGGCAATTGCTGGACT | 156bp |  |
| PhPDC2-R | TGGATCCCCATTCAAGTAGC |  |  |
| PhLDH-F | CAGTCCTTGCAAAGGGTTTC | 178bp |  |
| PhLDH-R | ATTCCCAATTGGTTTTGCAC |  |  |
| 26S RNA-F | AGCTCGTTTGATTCTGATTTCCAG | 185bp |  |
| 26S RNA-R | GATAGGAAGAGCCGACATCGAAGG |  |  |
| Bio-pADH1-2-F | Bio-TAAGCCTCACGGATCTAAGTCCCGCCCGAGAGTCT | 35bp | Wild-type and mutant probes synthesis used for EMSA |
| pADH1-2-R | AGACTCTCGGGCGGGACTTAGATCCGTGAGGCTTA |  |  |
| Bio-mpADH1-2-F | Bio-TAAGCCTCACGGAATTAAGTCCCGCCCGAGAGTCT | 35bp |  |
| mpADH1-2-R | AGACTCTCGGGCGGGACTTAATTCCGTGAGGCTTA |  |  |
| PhERF2-62-SK-F | ATGAATTCATGTGTGGTGGTGCTATAAT | 1159bp | Effector and reporter plasmid constructs used for dual luciferase assay |
| PhERF2-62-SK-R | ATGGTACCTTAGTAGACACCTCCCATCA |  |  |
| pADH1-2-LUC-F | ATGTCGACATAAAATAGCATAATGAGCC | 1272bp |  |
| pADH1-2-LUC-R | ATGGATCCGTCACTTGGTTTACAAAACC |  |  |
